# Expression Analyses of Rich2/Arhgap44, a Rho Family GTPase-Activating Protein, during Mouse Brain Development

**DOI:** 10.1159/000529051

**Published:** 2023-01-11

**Authors:** Naoki Goto, Masashi Nishikawa, Hidenori Ito, Mariko Noda, Nanako Hamada, Hidenori Tabata, Makoto Kinoshita, Koh-ichi Nagata

**Affiliations:** ^a^Division of Biological Science, Nagoya University Graduate School of Science, Nagoya, Japan; ^b^Department of Molecular Neurobiology, Institute for Developmental Research, Aichi Developmental Disability Center, Kasugai, Japan; ^c^Department of Neurochemistry, Nagoya University Graduate School of Medicine, Nagoya, Japan

**Keywords:** Rich2/Arhgap44, GTPase-activating protein, Mouse tissues, Brain development

## Abstract

Rho family small GTPases, such as Rho, Rac, and Cdc42, play essential roles during brain development, by regulating cellular signaling and actin cytoskeletal reorganization. Rich2/Arhgap44, a Rac- and Cdc42-specific GTPase-activating protein, has been reported to be a key regulator for dendritic spine morphology and synaptic function. Given the essential roles of Rac and Cdc42 in brain development, Rich2 is supposed to take part in brain development. However, not only the molecular mechanism involved but also the expression profile of Rich2 during neurodevelopment has not yet been elucidated. In this study, we carried out expression analyses of Rich2 by focusing on mouse brain development. In immunoblotting, Rich2 exhibited a tissue-dependent expression profile in the young adult mouse, and the expression was increased during brain development. In immunohistochemical analyses, Rich2 was observed in the cytoplasm of cortical neurons at postnatal day (P) 0 and then came to be enriched in the nucleus with moderate distribution in neuropils at P7. Later at P30, a complex immunostaining pattern of Rich2 was observed; Rich2 was distributed in the nucleus, cytoplasm, and neuropils in many cortical neurons, whereas other neurons frequently displayed little expression. In the hippocampus at P7, Rich2 was distributed mainly in the cytoplasm of excitatory neurons in the cornu ammonis regions, while it was moderately detected in the nucleus in the dentate granule cells. Notably, Rich2 was distributed in excitatory synapses of the cornu ammonis 1 region at P30. Biochemical fractionation analyses also detected Rich2 in the postsynaptic density. Taken together, Rich2 is found to be expressed in the central nervous system in a developmental stage-dependent manner and may be involved in synapse formation/maintenance in cortical neurons.

## Introduction

Rho family GTPases are known to play key roles in actin cytoskeletal reorganization during brain development; the most characterized members, RhoA, Rac1, and Cdc42, regulate distinct actin filament-containing structures crucial for neuronal migration, axonogenesis, dendritogenesis, and synaptic formation [1, 2]. Rho induces stress fiber assembly, and Rac regulates the formation of membrane ruffles (lamellipodia), while Cdc42 triggers filopodia formation. Similar to other GTPases, Rho family members function as binary switches that cycle between inactive (GDP-bound) and active (GTP-bound) conformational states [1, 3]. Interconversion of the 2 forms is spatiotemporally controlled by various regulatory molecules including guanine nucleotide exchange factors (GEFs) and GTPase-activating proteins (GAPs). GEFs facilitate GDP-release and GTP-loading to activate GTPases, while GAPs enhance intrinsic GTP-hydrolysis to inactivate them [4]. Given that the triad of GEF–GTPase–GAP is thought to play an essential role in a variety of neuronal functions, disruption of this triad is supposed to be responsible for neurodevelopmental and psychiatric disorders.

RhoGAP interacting with CIP4 homologs 2 (Rich2)/Arhgap44 is a Rac/Cdc42-specific GAP highly expressed in the central nervous system [5]. Rich2 has been reported to be localized at dendritic spines, control spine morphogenesis via regulation of Rac1, and be involved in the long-term increase in glutamatergic synaptic transmission efficiency associated with learning and memory [6, 7, 8]. However, not only the detailed role of Rich2 in brain development but also its tissue expression profile remained unknown. In the present study, we performed expression analyses of Rich2 with biochemical and morphological methods by focusing on mouse brain development. Consequently, Rich2 was found to be expressed in the cerebral cortex and hippocampus in developmental stage-dependent manners and was possible to be involved in synaptic functions of excitatory neurons.

## Materials and Methods

### Plasmids

Mouse *Rich2* was purchased from Genscript (Piscataway, NJ, USA) and cloned into pCAG-Myc vector. The following target sequence in *Rich2* was inserted into pSuper-puro RNAi vector (Cat# VEC-PBS-0008, OligoEngine, Seattle, WA, USA): GGAAATTCCAAATATTCAA (388–406, sh-Rich2‣1) and CATAACAACATCAAATACT (1121–1139, sh-Rich2‣2). Numbers indicate the positions from translational start sites. As a control RNAi vector, we used pSuper-Luc (sh-CTRL) designed against luciferase, CGTACGCGGAATACTTCGA (155–173) [9]. Glutathione-S-transferase (GST)-fused C-terminal fragment of Rich2 (aa 422–809; GST-Rich2-C) containing the epitope of anti-Rich2 (aa 465–492) was expressed in *E. coli*, purified, and used for the absorption experiment. All constructs were verified by DNA sequencing.

### Antibodies

The following monoclonal mouse antibodies were used: anti-Rich2 (Cat# sc-390609, Santa Cruz Biotechnology, CA, USA), anti-β-tubulin (Sigma-Aldrich, Cat# SAB4200715, RRID: AB_2827403), and anti-Myc (Cat# M047-3, RRID: AB_591112; Medical & Biological Laboratories, Nagoya, Japan).

### Preparation of Extracts from Mouse Tissues

Sample preparation was performed as described [10]. Briefly, tissues from ICR mice at P40 (Japan SLC, Shizuoka, Japan) were homogenized with the lysis buffer (50 mM Tris-HCl, pH 7.5, 0.1% NaF, 5 mM EDTA, 1 mM Na_3_VO_4_, and the protease inhibitor mix). Each suspension was sonicated on ice and centrifuged at 125,000 × *g* for 20 min at 4°C. The supernatants were used as cytosolic extracts. The pellets were sonicated in phosphate-buffered saline (PBS), washed once with PBS, centrifuged, and solubilized with the lysis buffer containing 2% SDS. Protein concentration was estimated with the BCA Protein Assay Reagent Kit (Pierce, Rockford, IL, USA) with bovine serum albumin (BSA) as a standard. SDS-PAGE (10% gel) and immunoblotting were conducted as described [10].

### Preparation of Postsynaptic Density

Sample preparation was performed as described [10]. Briefly, adult mouse brains were homogenized in 5 mM HEPES (pH 7.4) containing 1 mM MgCl_2_, 0.5 mM CaCl_2_, phosphatase inhibitors, and the protease inhibitor mix with a Teflon homogenizer. The resulting extract was centrifuged at 1,400 × *g* for 10 min to obtain S1 as a supernatant fraction. S2 fraction was then obtained by centrifugation of S1 at 13,800 × *g* for 10 min. The resulting pellet (P2) was resuspended in 6 mM Tris-HCl (pH 8.0) containing 0.32 M sucrose, loaded onto a discontinuous sucrose gradient, and centrifuged at 82,500 × *g* for 2 h. The synaptosome fraction was collected, extracted with 0.5% Triton X-100, and centrifuged at 32,800 × *g* for 20 min. The resulting pellet (PSD-I; crude PSD fraction) was again extracted with 0.5% Triton X-100 and centrifuged again at 201,800 × *g* for 1 h to obtain highly purified PSD fraction as a pellet (PSD-II). All procedures were performed at 4°C.

### Immunohistochemistry

Immunohistochemical analysis was carried out essentially as described [11]. Mouse brains of either sex were fixed by transcardial perfusion with 4% paraformaldehyde at P0, P7, and P30. Brains were removed and processed for paraffin embedding. Paraffinized coronal sections (6 μm thickness) were made at the level of the dorsal hippocampus and mounted on slide glasses. After deparaffinization, sections were treated with HistoVT one (Nacalai Tesque, Kyoto, Japan) for 20 min at 70°C for antigen retrieval and then incubated in methanol containing 3% hydrogen peroxide for 10 min for blocking nonspecific endogenous peroxidase activity. After blocking with TaKaRa Blocking Reagent A (Takara Bio, Otsu, Japan) for 1 h at room temperature (RT), samples were incubated with anti-Rich2 (1:100 dilution in 2% BSA/PBS) for 12 h at 4°C. After blocking with TaKaRa Blocking Reagent B (Takara Bio) for 1 h at RT, the slices were treated with secondary antibodies using TaKaRa POD Conjugate Set (Takara Bio) for 1 h at RT, and then subsequently treated with Takara DAB substrate (Takara Bio) for 10 min at RT. Images were captured using BZ-9000 microscope (Keyence, Osaka, Japan).

### Immunofluorescence Analyses

Immunohistochemical analysis was carried out essentially as described [10, 12]. Briefly, a frozen cortical section (14 μm thickness) was incubated in PBS containing 0.5% Triton X-100 and 0.1% BSA at RT for 1 h. Then, anti-Rich2 reaction was performed in PBS containing 0.05% Triton X-100 at 4°C overnight, followed by secondary antibody reaction (Alexa Fluor 488-labeled IgG) at RT for 1 h. Nuclei were visualized by *4*′*,6*-Diamino-*2*-phenylindole (DAPI) (Nacalai Tesque, Kyoto, Japan). Fluorescence signals were detected with an FV-3000 confocal laser microscope (Olympus, Tokyo, Japan).

### Cell Culture and Transfection

COS7 cells were cultured as described [13, 14]. Transient transfection was carried out using polyethylenimine “MAX” reagent (Polysciences, Warrington, PA, USA).

## Results

### Characterization of Anti-Rich2 Antibody

**Fig. 1:**
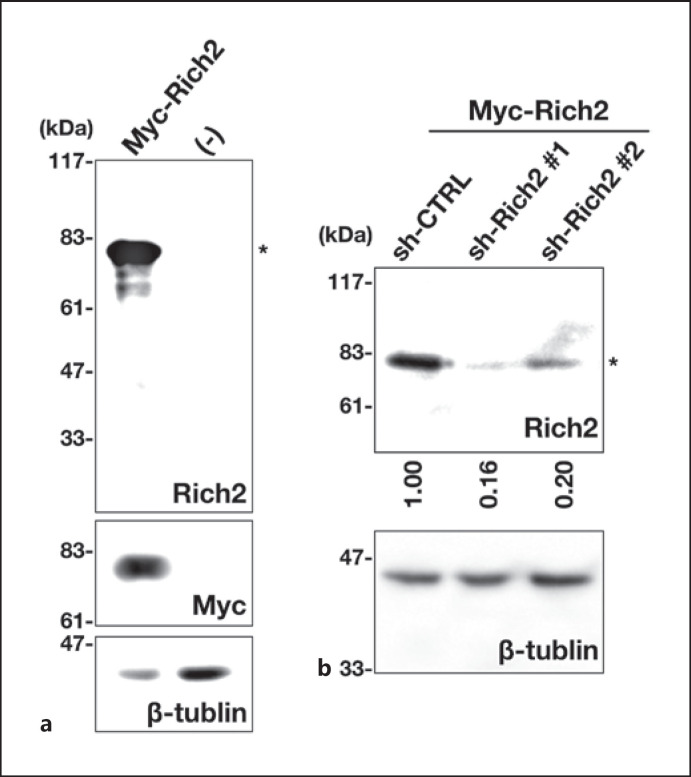
Characterization of an antibody for Rich2. **a** Lysates from COS7 cells expressing pCAG-Myc-Rich2 (0.2 μg) were subjected to SDS-PAGE (10% gel) followed by immunoblotting with antibodies for Rich2 (*top* panel), Myc-tag (*middle* panel), or β-tubulin (*bottom* panel). Molecular size marker was shown at *left*. **b** COS7 cells were transfected with sh-CTRL (control), sh-Rich2‣1, or sh-Rich2‣2 (1.0 μg each) together with pCAG-Myc-Rich2 (0.05 μg). After 24 h, the lysates were subjected to immunoblotting with anti-Rich2 (*upper* panel). The blot was reprobed with anti-β-tubulin for the loading control (*lower* panel). Molecular size markers were shown at *left*. Relative band intensity of Rich2 was calculated with ImageJ software based on densitometry, normalized against β-tubulin, and shown when the value of sh-CTRL was taken as 1.0. The data shown are representative of 3 independent experiments with very similar results. Asterisks (*) indicate the Rich2 band.

We first characterized the quality of anti-Rich2 used in this study. In immunoblotting analysis, anti-Rich2 recognized Myc-Rich2 with a molecular mass of ∼80 kDa expressed in COS7 cells (Fig. 1a, *upper* and *middle* panels). Immunoreactivity of anti-Rich2 was significantly reduced when Myc-Rich2 expression was silenced by RNAi vectors, sh-Rich2‣1 and ‣2 (Fig. 1b, *upper* panel), indicating specificity of the antibody. β-tubulin was visualized as a loading control (Fig. 1a, b, *lower* panels). From these results, anti-Rich2 was considered to recognize Rich2 specifically.

### Distribution of Rich2 in Young Adult Mouse Tissues

**Fig. 2:**
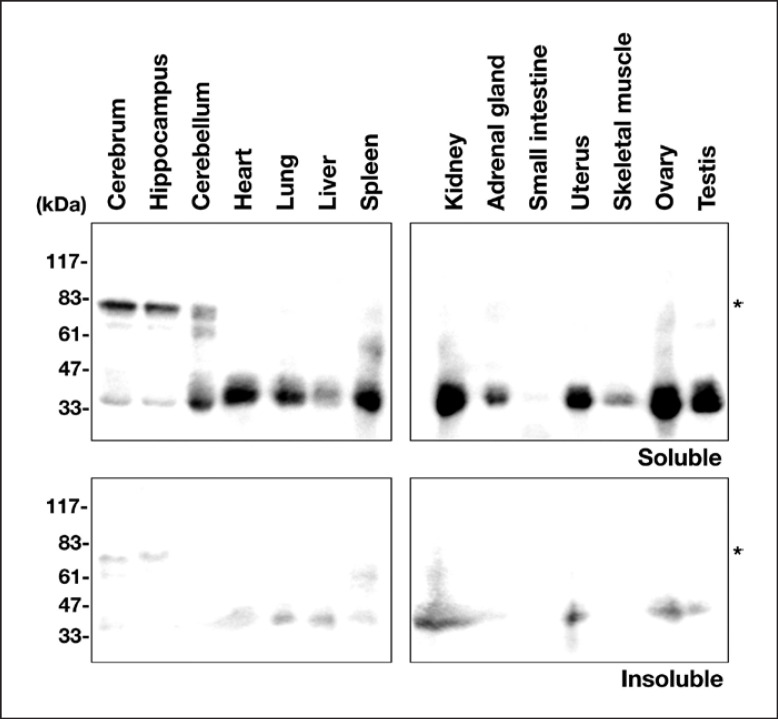
Tissue distribution of endogenous Rich2. Cytosolic and insoluble membrane fractions (20 μg of protein per lane) from adult mouse organs were subjected to immunoblotting with anti-Rich2. Molecular size markers were shown at the *left* of each panel. Asterisks (*) indicate the Rich2 band with a theoretical molecular mass.

The expression profile of Rich2 was analyzed by immunoblotting using the cytosolic and membrane fractions from a variety of tissues. In the soluble cytosolic fractions, Rich2 with a reasonable molecular mass of ∼80 kDa was observed as a major protein in the cerebrum and hippocampus (Fig. 2, *upper* panels). On the other hand, a protein band with ∼35 kDa was strongly detected in the cytoplasm of many non-neuronal tissues and the cerebellum. While this protein might be a yet unidentified isoform, the possibility cannot be ruled out that this is a degradation product during sample preparation or a non-specific band. Further genetic analyses of Rich2 are required to identify its isoforms. As to the insoluble membrane fractions, Rich2 was only faintly visualized in many tissues tested (Fig. 2, *lower* panels). From these data, we concluded that Rich2 with an appropriate molecular mass is expressed mainly in the cytoplasm of the cerebrum and hippocampus.

### Distribution of Rich2 in the Mouse Brain

**Fig. 3:**
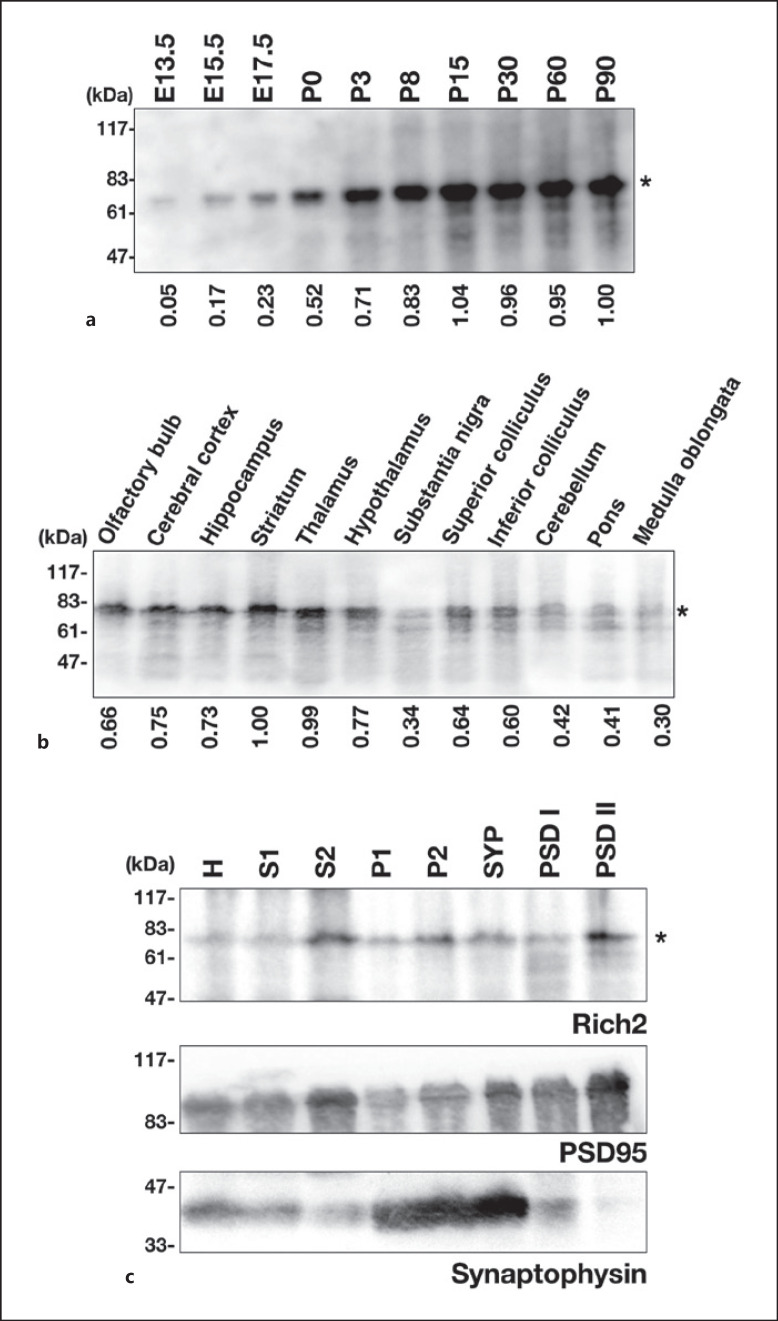
Analyses of endogenous Rich2 expression in the mouse brain. **a** Whole brain extracts (20 μg of protein per lane) at various developmental stages were immunoblotted with anti-Rich2. The expression level of Rich2 was measured using ImageJ software, and relative expression levels were shown as fold-increase over the expression level at P90. The data shown are representative of 3 independent experiments with very similar results. **b** Whole extracts from dissected brain regions (30 μg of protein per lane) were analyzed in immunoblotting with anti-Rich2. The expression level was measured as in (**a**). The data shown is representative of 3 independent experiments with similar results. **c** Aliquots of the brain fractions (30 μg of protein per lane) were immunoblotted with anti-Rich2 (*top* panel). The blot was reprobed with anti-PSD95 (*middle* panel) or anti-synaptophysin (*bottom* panel). H, homogenate; S1, soluble fraction; S2, crude cytosol fraction; P1, nuclear fraction; P2, crude membrane fraction; SYP, synaptosomal fraction; PSD-I, PSD fraction-I; PSD-II, PSD fraction-II. Asterisks (*) indicate the Rich2 band.

To gain some insight into the involvement of Rich2 in neuronal development, we analyzed the Rich2 expression in whole brain extracts prepared at various developmental stages. Rich2 with a molecular mass of ∼80 kDa was found to be expressed at E13.5, then gradually increased, reached a maximum at P15, and plateaued thereafter (Fig. 3a). To further characterize the expression profile of Rich2 in the brain, 12 regions were dissected from a young adult mouse. Immunoblotting revealed that the olfactory bulb, cerebral cortex, hippocampus, striatum, and thalamus contain relatively high amount of Rich2, while weak expression was observed in the hypothalamus and superior and inferior colliculi (Fig. 3b). When biochemical subcellular fractions were prepared from mouse brains and subjected to immunoblotting, Rich2 was at least partially enriched in the PSD-II fractions (Fig. 3c, *top* panel). Expression profiles of anti-synaptophysin and anti-PSD95 indicated that the fractionation was carried out appropriately (Fig. 3c, *middle* and *bottom* panels). Although the physiological function of Rich2 during corticogenesis is enigmatic, the obtained results may support the role of Rich2 in the synapse function [5].

### Immunohistochemical Analyses of Rich2 in the Mouse Cerebral Cortex and Hippocampus

**Fig. 4:**
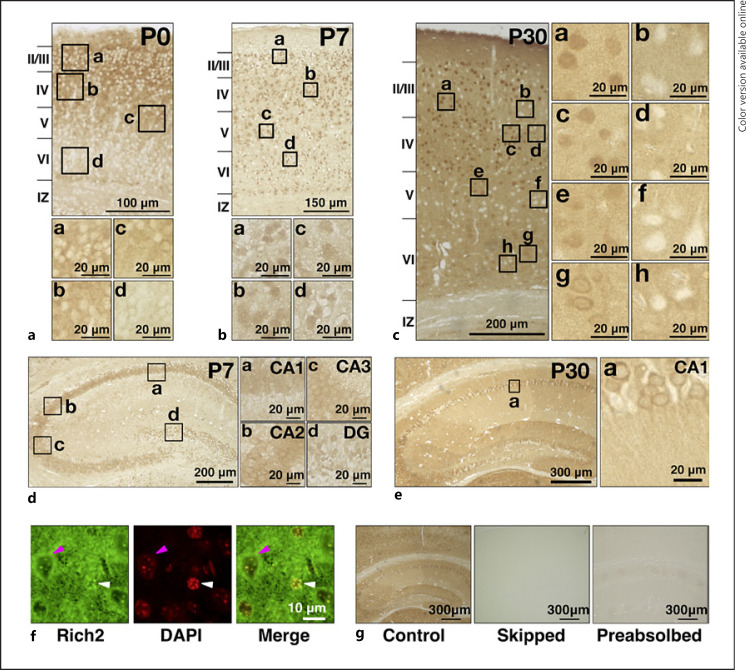
Immunohistochemical and immunofluorescent analyses of Rich2 in the cerebral cortex and hippocampus. **a**−**c** Coronal paraffin sections of the cerebral cortex at P0 (**a**), P7 (**b**), and P30 (**c**) were stained with anti-Rich2. Boxed areas in the *top* panels of (**a, b**) and in the *left* panel of (**c**) were magnified. Layers II–VI, the intermediate zone (IZ), and the white matter (WM) were indicated. **d, e** Images of the hippocampus were shown at P7 (**d**) and P30 (**e**). Boxed areas indicated in the *left* images of (**d, e**) were magnified. **f** Immunofluorescent analysis staining a cortical slice (P30) with anti-Rich2 (**a**). Nuclei were stained with DAPI (**b**). White and magenta arrowheads indicate nuclear enrichment and perinuclear distribution, respectively, of Rich2. Merged image was also shown (**c**). **g** Staining of cortical sections (P30) was conducted with anti-Rich2 (**a**), without the anti-Rich2 reaction step (**b**), or with anti-Rich2 preabsorbed with GST-Rich2-C (**c**), respectively. CA, cornu ammonis; DG, dentate gyrus.

Given the expression in a developmental stage-dependent manner in the brain, Rich2 is supposed to play a role in neuronal development. To further look into the involvement of Rich2 in neuronal development and differentiation, we carried out immunohistochemical analyses with the cerebral cortex at P0, P7, and P30. At P0, Rich2 was distributed in the cytoplasm of cortical neurons mainly in layers II–V (Fig. 4a). Then, nuclear staining of Rich2 became prominent throughout the cortex at P7 (Fig. 4b). Later at P30, a complex immunostaining pattern of Rich2 was observed; Rich2 was distributed in the nucleus and cytoplasm in many cortical neurons, whereas other neurons frequently displayed little expression (Fig. 4c). Rich2 was also diffusely distributed in neuropils but not in the white matter (Fig. 4c). Rich2 was also expressed in the hippocampus with distribution in the cornu ammonis (CA) subregions and dentate gyrus at P7 (Fig. 4d). When magnified, Rich2 was mainly distributed in the cytoplasm of pyramidal neurons in the CA1–CA3 at P7 (Fig. 4d, a-c). On the other hand, Rich2 was relatively enriched in the nucleus in the dentate gyrus at this time (Fig. 4d, d). Later at P30, the molecule showed moderate expression in neuropils, suggesting the enrichment of Rich2 at synapses (Fig. 4e, a). Notably, Rich2-mRNA expression profile is available for an adult mouse online (https://mouse.brain-map.org/gene/show/85063), which appears to be consistent with the immunohistochemical data. We then confirmed the nuclear localization of Rich2 by co-staining with a DNA marker, DAPI. When the cortical layer VI of the P30 brain was analyzed, Rich2 was relatively enriched in the nucleus in some neurons (Fig. 4f, *white arrowhead*), while perinuclear distribution was observed in some other cells (Fig. 4f, *magenta arrowhead*). Finally, specificity of anti-Rich2 was validated; the immunoreactivity became very weaker when the step of anti-Rich2 incubation was skipped in the staining procedure (Fig. 4g, a, b) or anti-Rich2 was pretreated with an excess amount of the GST-Rich2 fragment containing the epitope (Fig. 4g, a, c).

## Discussion

In the present study, we performed biochemical and immunohistochemical characterization of Rich2 during mouse brain development, by focusing on the cerebral cortex and hippocampus. Since the molecular weight of Rich2 transcript variant 1 (NM_001099288.1, 808 amino acid) is calculated to be 88,270, the ∼80 kDa protein observed in western blotting analyses (Fig. 1, 2, 3) may be a major isoform of mouse Rich2. On the other hand, although the ∼35 kDa band observed in various tissues is possible to be a yet unidentified isoform (Fig. 2), its molecular identity remains to be elucidated. Considering that structural analysis of the mouse *Rich2* gene has not been conducted thoroughly, further genetic analyses as well as preparation of isoform-specific antibodies should be required to identify this protein.

Biochemical fractionation revealed partial localization of Rich2 in the PSD fraction from the adult mouse cerebral cortex (Fig. 3c). Together with the results that the expression level was increased during brain development (Fig. 3b), Rich2 is possible to be involved in synaptic functions in differentiated cortical neurons. Consistently, Rich2 was distributed in the neuropil of the adult mouse cerebral cortex and hippocampus in immunohistochemical analyses (Fig. 4). Thus, Rich2 is supposed to play crucial roles in both synaptogenesis and the synapse function through the regulation of Rac and/or Cdc42 signaling. This hypothesis is consistent with the results reported previously that spatiotemporal regulation of Rac1 activity is essential for the various events in neurodevelopment, such as neuronal migration, dendritogenesis, axonogenesis, and spine morphogenesis [2, 15, 16]. Based on its critical role during cortical development, Rac3 is also possible to participate in the Rich2-dependent cellular processes during and after corticogenesis [13]. In this context, Rich2-knockout mice demonstrated impaired motor learning, increased stereotyped self-grooming, and decreased exploration [7]. It is notable that Rich2 was hardly detected in a considerable number of excitatory neurons in the cerebral cortex and hippocampus of the young adult mouse (Fig. 4c, e). Although physiological relevance of this selective expression remains to be clarified, other Rac/Cdc42-GAP(s) may function in a complementary manner.

The observations in this study support the hypothesis that Rich2 is involved in cortical and hippocampal development as well as synaptic functions in differentiated neurons. Further cell biological investigation with anti-Rich2 is crucial to elucidate the physiological role of Rich2 in brain development and functions.

## Conclusion

In the present study, we found developmental stage-dependent expression of Rich2 in the mouse brain using anti-Rich2. Immunohistochemical analyses revealed that Rich2 was expressed in the cerebral cortex and hippocampus in spatiotemporal manners. Rich2 was detected in the PSD fraction in biochemical fractionation. These results suggest that Rich2 is essential for neural network formation and maintenance in the cerebral cortex and hippocampus.

## Statement of Ethics

We followed the fundamental guidelines for proper conduct of animal experiments and related activity in academic research institution under the jurisdiction of the Ministry of Education, Culture, Sports, Science and Technology, Japan. All protocols for animal handling and treatment were reviewed and approved by the Animal Care and Use Committee of Institute for Developmental Research, Aichi Developmental Disability Center (approval number; 2019-013).

## Conflict of Interest Statement

All authors have no conflict of interest.

## Funding Sources

This work was supported in part by Japan Society for the Promotion of Science (JSPS) KAKENHI Grant-in-Aid for Scientific Research (B) (grant number JP19H03629), Grant-in-Aid for Scientific Research (C) (grant number JP19K07059), Grant-in-Aid for Early-Career Scientists (grant number JP21K15895), and a grant-in-aid of the Practical Research Project for Rare/Intractable Diseases from Japan Agency for Medical Research and Development (AMED) (15ek0109040h0002).

## Author Contributions

Naoki Goto and Masashi Nishikawa performed biochemical and immunofluorescence analyses and wrote the manuscript. Mariko Noda performed biochemical fractionation. Hidenori Ito, Nanako Hamada, and Hidenori Tabata supervised immunofluorescence analyses and helped with the interpretation of the data. Masashi Nishikawa, Hidenori Ito, and Koh-ichi Nagata conceived and designed the experiment. Makoto Kinoshita and Koh-ichi Nagata helped to draft the manuscript. All authors read and approved the final manuscript.

## Data Availability Statement

All data generated or analyzed during this study are included in this article. Further inquiries can be directed to the corresponding author.
